# Risk factors for breast cancer characterized by the estrogen receptor alpha A908G (K303R) mutation

**DOI:** 10.1186/bcr1731

**Published:** 2007-06-06

**Authors:** Kathleen Conway, Eloise Parrish, Sharon N Edmiston, Dawn Tolbert, Chiu-Kit Tse, Patricia Moorman, Beth Newman, Robert C Millikan

**Affiliations:** 1 Department of Epidemiology, School of Public Health, CB 7435, University of North Carolina, Chapel Hill, NC 27599, USA; 2 Lineberger Comprehensive Cancer Center, School of Medicine, CB 7295, University of North Carolina, Chapel Hill, NC 27599, USA; 3 Department of Community and Family and Preventive Medicine, Duke University School of Medicine, Box 2949, Durham, NC 27710, USA; 4 School of Public Health, Institute for Health and Biomedical Innovation, Queensland University of Technology, Brisbane 4059, Australia

## Abstract

**Introduction:**

Estrogen is important in the development of breast cancer, and its biological effects are mediated primarily through the two estrogen receptors alpha and beta. A point mutation in the estrogen receptor alpha gene, *ESR1*, referred to as A908G or K303R, was originally identified in breast hyperplasias and was reported to be hypersensitive to estrogen. We recently detected this mutation at a low frequency of 6% in invasive breast tumors of the Carolina Breast Cancer Study (CBCS).

**Methods:**

In this report, we evaluated risk factors for invasive breast cancer classified according to the presence or absence of the *ESR1 *A908G mutation in the CBCS, a population-based case-control study of breast cancer among younger and older white and African-American women in North Carolina. Of the 653 breast tumors evaluated, 37 were *ESR1 *A908G mutation-positive and 616 were mutation-negative.

**Results:**

*ESR1 *A908G mutation-positive breast cancer was significantly associated with a first-degree family history of breast cancer (odds ratio [OR] = 2.69, 95% confidence interval [CI] = 1.15 to 6.28), whereas mutation-negative breast cancer was not. Comparison of the two case subgroups supported this finding (OR = 2.65, 95% CI = 1.15 to 6.09). There was also the suggestion that longer duration of oral contraceptive (OC) use (OR = 3.73, 95% CI = 1.16 to 12.03; *P*_trend _= 0.02 for use of more than 10 years) and recent use of OCs (OR = 3.63, 95% CI = 0.80 to 16.45; *P*_trend _= 0.10 for use within 10 years) were associated with *ESR1 *A908G mutation-positive breast cancer; however, ORs for comparison of the two case subgroups were not statistically significant. Hormone replacement therapy use was inversely correlated with mutation-negative breast cancer, but the effect on mutation-positive cancer was unclear due to the small number of postmenopausal cases whose tumors carried the mutation. Mutation-negative breast cancer was associated with several reproductive factors, including younger age at menarche (OR = 1.46, 95% CI = 1.09 to 1.94) and greater total estimated years of ovarian function (OR = 1.82, 95% CI = 1.21 to 2.74).

**Conclusion:**

These preliminary results suggest that OCs may interact with the *ESR1 *A908G mutant receptor to drive the development of some breast tumors.

## Introduction

Most major risk factors for breast cancer are hormonal or reproductive factors that increase exposure to estrogen and/or progesterone [1]. The importance of estrogen in breast cancer development is also supported by studies demonstrating the occurrence of marked changes in estrogen signaling and in the expression of the two estrogen receptors (ERs), ER alpha and ER beta, during breast tumorigenesis and progression [2-8].

Although mutations in the gene encoding ER alpha, *ESR1*, are uncommon in primary breast tumors [3], a specific point mutation that occurs at nucleotide 908 within codon 303 and that is referred to as A908G or K303R was described several years ago by Fuqua and colleagues [9] in one third of typical breast hyperplasias. The A908G mutation affects the border of the hinge and the hormone-binding domains of *ESR1 *and results in an amino acid change of lysine to arginine (K303R). Compared with the wild-type receptor, the A908G mutant exhibited hypersensitivity to estrogen and was associated with increased cellular proliferation at sub-physiologic levels of estrogen [9]. The A908G mutant receptor displayed similar affinity for estradiol as wild-type receptor but showed enhanced binding to the TIF-2 (transcription intermediary factor-2) coactivator at low hormone levels [9]. More recent studies have also shown that the *ESR1 *A908G mutation in codon 303 increases phosphorylation at the Ser305 residue through the P13 kinase/Akt signaling cascade [10], protein kinase A [11], and p21-activated kinase [10], but the downstream functional effects of this phosphorylation remain unclear.

We recently detected the *ESR1 *A908G mutation at a low frequency of 6% in the primary invasive breast tumors of the Carolina Breast Cancer Study (CBCS), a population-based case-control study of mostly early stage breast cancer in North Carolina [12]. This mutation was confirmed to be somatic in nature and not a germline variant. Mutation-positive tumors were more likely to have mixed lobular/ductal histology and combined grade II (versus grade I) compared with mutation-negative tumors.

The presence of the *ESR1 *A908G mutation in both breast hyperplasias and invasive carcinomas suggests that it may be an early genetic defect present in the breast tissue of some women and that it, alone or in conjunction with environmental factors, may help to drive the development of some breast tumors. The existence of distinct subtypes of breast cancer as defined by differences in gene expression profiles and in expression of ER and other biomarkers has now been clearly established [13,14]. These subtypes vary in their clinical prognosis and may also be characterized by differences in risk factor profiles. Given the reported hypersensitivity of the mutant *ESR1 *A908G mutant receptor to estrogen, it was of interest to determine whether hormonal risk factors in the CBCS might be associated with breast cancer characterized by the presence or absence of the *ESR1 *A908G mutation. In particular, we evaluated exposure to exogenous hormones, such as oral contraceptives (OCs) or hormone replacement therapy (HRT), as well as reproductive factors linked to greater endogenous estrogen and progesterone exposure. Our findings suggest that OC use and first-degree family history of breast cancer may be associated with *ESR1 *A908G mutation-positive breast cancer, whereas reproductive factors associated with longer exposure to endogenous hormones may be related to mutation-negative breast cancer.

## Materials and methods

### Study population

This study used subjects and data from phase 1 of the CBCS, a population-based, case-control study of invasive breast cancer conducted among African-American and white women (age range, 20 to 74 years) residing in a 24-county area in central and eastern North Carolina [15]. Both cases and controls were sampled using a modification of randomized recruitment [15]. Sampling probabilities ensured approximately equal samples among cases and controls in the four age-race groups: younger (age range, 20 to 49 years) African-American women, older (age range, 50 to 74 years) African-American women, younger white women, and older white women. Details of recruitment of participants and response rates have been published previously [15]. Incident cases of breast cancer diagnosed between 1 May 1993 and 30 May 1996 were eligible as cases and were identified using the North Carolina Central Cancer Registry's Rapid Case Ascertainment System; of these, 861 patients with breast cancer consented to participate in the CBCS. Clinical data and information on tumor characteristics were obtained from medical records or direct histopathologic review of tumor tissue as described [14]. Controls were drawn from North Carolina Division of Motor Vehicle lists for women ages 20 to 64 years and US Health Care Financing Administration lists for women ages 65 to 74 years and were frequency matched to cases by race and age (in 5-year age categories). A total of 790 controls were eligible for and consented to participate in the CBCS. All aspects of this research were approved by the Institutional Review Board of the University of North Carolina School of Medicine.

### Tumor tissues and analysis of the *ESR1 *A908G mutation

Formalin-fixed paraffin-embedded (FFPE) tumor blocks were obtained from pathology departments at participating hospitals for 798 of the 861 breast cancer cases. Of these, 684 were of sufficient size to allow for the sectioning of 10-μm-thick tissue sections for molecular analyses. Tumors were sectioned and underwent standardized histopathologic review as previously described [16]. With the hematoxylin-and-eosin-stained slide as a guide, the area of tumor was microdissected away from other surrounding non-tumor tissue, and DNA lysates were prepared using proteinase K extraction.

Of the 684 tumors available for molecular studies, 653 were successfully screened for mutations in a 104-base pair region of exon 4 surrounding codon 303 of *ESR1 *by using a combination of SSCP (single-strand conformational polymorphism) and phosphorus-33-cycle DNA sequencing as described previously [14]. The tumors that were evaluated for the *ESR1 *A908G mutation were more likely to be later stage (*P *= 0.005), of larger size (*P *= 0.0002), and lymph node-positive (*P *= 0.006) and to exhibit higher combined grade (*P *= 0.04) than tumors that were not screened, consistent with the greater availability of tumor tissue from larger breast tumors. However, the cases screened for mutations did not differ from those that were not screened based on age (*P *= 0.42), menopausal status (*P *= 0.90), race (*P *= 0.63), ER status (*P *= 0.68), or tumor histology (*P *= 0.48).

### Risk factor information

Data were obtained from cases and controls during in-person interviews conducted by female registered nurses. The nurse-interviewer elicited information on demographics and potential breast cancer risk factors. Interviews were completed for 77% (*n *= 861) of eligible and locatable cases and for 68% (*n *= 790) of eligible and locatable controls. The nurses drew a blood sample and measured weight, height, and waist and hip circumferences at the time of interview. Age was based on age at diagnosis in cases or on age at selection in controls. Race was classified according to self-report. Fewer than 2% of the participants described themselves as races other than African-American or white; these subjects were categorized with African-American women for statistical analysis. Women who used OCs or HRT for 3 months or longer were considered ever users. Use of HRT was evaluated among postmenopausal women.

Menopausal status was determined by information provided by participants in the interview. Women who were 50 years of age or older at the time of interview were considered to be postmenopausal if their periods had stopped naturally, due to surgery (hysterectomy and/or bilateral oophorectomy), or due to chemotherapy or radiation (unrelated to the present diagnosis of breast cancer). Women who were less than 50 years of age were considered postmenopausal if their periods stopped due to natural menopause, bilateral oophorectomy, radiation, or chemotherapy. All other women were classified as premenopausal.

### Statistical methods

To quantify the associations between reproductive, hormonal, and other risk factors and breast cancer subtype defined by *ESR1 *908G mutation status, odds ratios (ORs) and 95% confidence intervals (CIs) comparing each case subgroup to controls were calculated. The ORs for breast cancer were calculated using unconditional logistic regression as implemented in the SAS software program (version 8.2; SAS Institute Inc., Cary, NC, USA). Offset terms were incorporated into models using the SAS procedure, PROC GENMOD, to account for the sampling probabilities used to define eligible cases and controls [15]. Potential confounding was evaluated by including covariates in multivariate models, and these were selected based on their role in matching or their previously demonstrated relevance to breast cancer risk. These included age, race, menopausal status, family history of breast cancer in a first-degree relative, age at menarche, age at first full-term pregnancy (AFFTP) and parity incorporated into a composite variable (nulliparous, parity ≥ 1 and AFFTP = 26 years, parity = 1 and AFFTP < 26 years, parity ≥ 2/AFFTP ≥ 26 years, and parity ≥ 2 and AFFTP < 26 years), lactation (ever, never), duration of ovarian function defined as the number of years between onset of menarche and menopause (natural or otherwise), smoking, alcohol consumption (ever, never), and body mass index (BMI) (less than 25, 25 to less than 30, or greater than or equal to 30 kg/m^2^). Ever smoking was defined as lifetime exposure to at least 100 cigarettes. Trend tests for risk factors with ordinal levels (for example, such as duration of OC use) were performed, and *P *values were calculated using Wald χ^2 ^statistics.

## Results

### Characteristics of cases and controls

The characteristics of cases and controls in the CBCS have been described previously [17]. Slightly more than half of the subjects were younger than 50 years of age, approximately half were premenopausal, and approximately 40% were African-American. Cases were significantly more likely than controls to have a first-degree family history of breast cancer.

For the present study, a total of 653 FFPE invasive breast tumors from cases in the CBCS were screened for mutations in a 104-base pair region of exon 4 surrounding codon 303 of *ESR1*. The clinical characteristics of cases evaluated for the *ESR1 *mutation have been reported previously [14]. The majority (88%) of cases had stage 1 or 2 disease, 60% were node-negative, 59% were ER-positive, and 79% were diagnosed with invasive ductal carcinoma. Of the 653 breast cancer cases screened, 37 (5.7%) were positive for the *ESR1 *A908G mutation. When analyzed according to *ESR1 *A908G mutation status, tumors that were mutation-positive were of somewhat larger size (*P *= 0.11), were of somewhat higher combined grade (*P *= 0.03 grade II versus grade I), and were more likely to have mixed lobular/ductal histology (*P *= 0.10). However, there was no difference in ER protein expression between mutation-positive and mutation-negative breast tumors (*P *= 0.57).

### Risk factors for breast cancer according to *ESR1 *A908G mutation status

Table 1 presents both minimally adjusted and more fully adjusted ORs and 95% CIs comparing *ESR1 *mutation-positive cases (*n *= 37) and mutation-negative cases (*n *= 616) to controls (*n *= 790). Both mutation-positive and mutation-negative cases were less likely than controls to have ever breast fed, but this inverse association was more pronounced for mutation-positive cases (OR = 0.29, 95% CI = 0.12 to 0.71) than for mutation-negative cases (OR = 0.69, 95% CI = 0.53 to 0.89). Mutation-negative cases were more likely than controls to have younger age at menarche (OR = 1.46, 95% CI = 1.09 to 1.94) and longer duration of ovarian function if postmenopausal (OR = 1.82, 95% CI = 1.21 to 2.74 for 36 years or more; *P*_trend _= 0.004). BMI overall was not associated with either mutation-positive or mutation-negative cancer; stratification on menopausal status yielded numbers of mutation-positive cases that were too sparse to analyze. Adjustment for additional covariates did not appreciably alter the ORs.

**Table 1:** Associations between reproductive and other risk factors and breast cancer characterized by *ESR1 *A908G mutation status

**Risk factor**	**Controls (*n *= 790)**		**Mutation-positive cases (*n *= 37)**						**Mutation-negative cases (*n *= 616)**					
	***n***	**(%)**	***n***	**(%)**	**OR1**^a^	**95% CI**	**OR2**^b^	**95% CI**	***n***	**(%)**	**OR1**^a^	**95% CI**	**OR2**^b^	**95% CI**
**Age (years)**														
50+	383	(48.5)	19	(51.4)	^c^		^c^		246	(39.9)	^c^		^c^	
<50	407	(51.5)	18	(48.6)					370	(60.1)				
**Race**														
White	458	(58.0)	25	(67.6)	^c^		^c^		371	(60.2)	^c^		^c^	
African-American	332	(42.0)	12	(32.4)					245	(39.8)				
**Menopausal status**														
Postmenopausal	435	(55.1)	22	(59.5)	1.00	---	1.00^d^	---	295	(47.9)	1.00	---	1.00^d^	---
Premenopausal	355	(44.9)	15	(40.5)	1.27	0.40–4.05	1.63	0.47–5.64	321	(52.1)	1.08	0.78–1.50	1.12	0.80–1.58
**First-degree family history of BC**														
No	671	(88.3)	26	(72.2)	1.00	---	1.00^e^	---	523	(87.0)	1.00	---	1.00^e^	---
Yes	89	(11.7)	10	(27.8)	2.54	1.16–5.55	2.69	1.15–6.28	78	(13.0)	1.17	0.84–1.62	1.22	0.87–1.71
**First-degree family history of BC and/or ovarian cancer**														
No	664	(87.4)	24	(66.7)	1.00	---	1.00^e^	---	514	(85.5)	1.00	---	1.00^e^	---
Yes	96	(12.6)	12	(33.3)	3.13	1.49–6.58	3.48	1.56–7.76	87	(14.5)	1.21	0.88–1.66	1.27	0.92–1.76
**Prior benign breast biopsy**														
No	649	(82.4)	28	(80.0)	1.00	---	1.00	---	518	(84.2)	1.00	---	1.00	---
Yes	139	(17.6)	7	(20.0)	1.21	0.51–2.87	1.17	0.47–2.91	97	(15.8)	1.00	0.75–1.33	0.94	0.70–1.28
**BMI**														
<25	250	(32.2)	15	(41.7)	1.00	---	1.00^f^	---	224	(37.1)	1.00	---	1.00^f^	---
25 to <30	250	(32.2)	10	(27.8)	0.73	0.32–1.69	0.78	0.32–1.90	180	(29.8)	0.87	0.66–1.14	0.83	0.63–1.10
30+	277	(35.6)	11	(30.5)	0.80	0.34–1.89	0.76	0.29–1.98	200	(33.1)	0.85	0.64–1.12	0.80	0.60–1.08
**Smoking**														
Never	423	(53.5)	18	(48.7)	1.00	---	1.00^g^	---	322	(52.3)	1.00	---	1.00^g^	---
Ever	367	(46.5)	19	(51.3)	1.16	0.59–2.27	1.04	0.49–2.19	294	(47.7)	1.08	0.87–1.34	1.11	0.88–1.41
**Alcohol consumption**														
Never	231	(29.3)	14	(37.8)	1.00	---	1.00^h^	---	178	(28.9)	1.00	---	1.00^h^	---
Ever	558	(70.7)	23	(62.2)	0.79	0.39–1.63	0.74	0.32–1.71	438	(71.1)	0.95	0.75–1.22	0.88	0.67–1.16
**Age at menarche (years)**														
14+	203	(25.8)	10	(27.0)	1.00	---	1.00^i^	---	130	(21.1)	1.00	---	1.00^i^	---
13	206	(26.2)	9	(24.3)	0.96	0.38–2.44	1.22	0.43–3.41	165	(26.8)	1.28	0.95–1.75	1.38	1.01–1.91
≤12	378	(48.0)	18	(48.6)	1.12	0.50–2.50	1.42	0.57–3.53	321	(52.1)	1.36	1.04–1.79	1.46	1.09–1.94
**Parity**														
Nulliparous	89	(11.3)	5	(13.5)	1.00	---	1.00^j^	---	100	(16.2)	1.00	---	1.00^j^	---
Parous	701	(88.7)	32	(86.5)	0.93	0.34–2.54	1.30	0.44–3.85	516	(83.8)	0.76	0.55–1.04	0.92	0.65–1.29
**Age at first full-term pregnancy (years)**														
Nulliparous	89	(11.3)	5	(13.9)	1.00	---	1.00^j^	---	100	(16.3)	1.00	---	1.00^j^	---
10–19	236	(29.9)	8	(22.2)	0.74	0.23–2.39	1.03	0.28–3.79	181	(29.5)	0.82	0.57–1.16	0.99	0.67–1.46
20–24	265	(33.6)	11	(30.5)	0.82	0.27–2.52	1.14	0.35–3.74	181	(29.5)	0.73	0.51–1.03	0.88	0.60–1.27
25+	198	(25.2)	12	(33.3)	1.20	0.40–3.63	1.71	0.51–5.78	152	(24.8)	0.73	0.51–1.05	0.90	0.60–1.34
**Number full-term births**														
Nulliparous	89	(11.3)	5	(13.5)	1.00	---	1.00^j^	---	100	(16.2)	1.00	---	1.00^j^	---
1	141	(17.8)	5	(13.5)	0.74	0.20–2.69	0.99	0.26–3.78	117	(19.0)	0.79	0.54–1.17	0.89	0.59–1.34
2 or more	560	(70.9)	27	(73.0)	0.98	0.36–2.72	1.43	0.47–4.35	399	(64.8)	0.75	0.54–1.03	0.92	0.65–1.31
**Breastfeeding**														
Never	476	(60.3)	26	(70.3)	1.00	---	1.00^k^	---	413	(67.0)	1.00	---	1.00^k^	---
Ever	314	(39.7)	11	(29.7)	0.52	0.25–1.09	0.29	0.12–0.71	203	(33.0)	0.70	0.56–0.87	0.69	0.53–0.89
**Age at menopause (years) (postmenopausal)**														
<45	192	(45.6)	10	(45.5)	1.00	-	1.00^d^	---	117	(39.9)	1.00	---	1.00^d^	---
45–49	119	(28.3)	7	(31.8)	1.13	0.42–3.08	1.47	0.45–4.79	93	(31.7)	1.38	0.96–1.97	1.39	0.95–2.03
50+	110	(26.1)	5	(22.7)	0.82	0.27–2.53	1.32	0.36–4.77	83	(28.3)	1.39	0.95–2.03	1.50	1.00–2.24
**Duration of ovarian function (years) (postmenopausal)**														
≤29	139	(33.2)	6	(27.2)	1.00	---	1.00^d^	---	79	(27.0)	1.00	---	1.00^d^	---
30–35	141	(33.6)	10	(45.4)	1.66	0.58–4.74	1.78	0.49–6.46	98	(33.4)	1.33	0.90–1.95	1.32	0.88–1.99
36+	139	(33.2)	6	(27.2)	0.98	0.30–3.18	1.38	0.35–5.45	116	(39.6)	1.69	1.15–2.48	1.82	1.21–2.74
P_trend_						0.97		0.70				0.007		0.004

^a^OR1 minimally adjusted for age, race, and offsets.

^b^OR2 adjusted for age, race, offsets, menopausal status (except d), family history of breast cancer (except e), BMI (<25, 25 to <30, 30+) (except f), smoking status (never, former, current) (except g), alcohol intake (ever, never) (except h), age at menarche (<11, 11–12, 13, 14+) (except i), parity/age first full-term pregnancy (except j), and breast feeding (never, ever) (except ^k^).

^c^ORs were not calculated for age or race as these factors were part of the sampling scheme. BC, breast cancer; BMI, body mass index; CI, confidence interval; *ESR1*, estrogen receptor alpha; OR, odds ratio.

Compared with controls, *ESR1 *A908G mutation-positive cases were significantly more likely to have a first-degree family history of breast cancer (OR = 2.69, 95% CI = 1.15 to 6.28) or a first-degree family history of breast and/or ovarian cancer (OR = 3.48, 95% CI = 1.56 to 7.76), whereas mutation-negative cases did not (OR = 1.22, 95% CI = 0.87 to 1.71 for first-degree family history of breast cancer). Mutation-positive cases were also directly compared to the mutation-negative cases to determine the degree of risk heterogeneity between these two disease subtypes. First-degree family histories of breast cancer (OR = 2.65, 95% CI = 1.15 to 6.09) or of breast and/or ovarian cancer (OR = 3.15, 95% CI = 1.44 to 6.91) were both significantly associated with *ESR1 *A908G mutation positivity (not shown).

As shown in Table 2, multivariate logistic regression analyses indicated that *ESR1 *mutation-positive breast cancer cases were more likely to have ever used OCs (OR = 1.72, 95% CI = 0.66 to 4.44), but this result was not statistically significant. An association with OC use was strongest among long-term users (OR = 3.73, 95% CI = 1.16 to 12.03 for duration more than 10 years) and recent users (OR = 3.63, 95% CI = 0.80 to 16.45 for use within 10 years). Women who had used OCs for more than 10 years and within the past 10 years showed the greatest risk of *ESR1 *mutation-positive breast cancer (OR = 6.49, 95% CI = 1.32 to 31.89).

**Table 2:** Associations between OC or HRT use and breast cancer defined by *ESR1 *A908G mutation status

**Risk factor**	**Controls (*n *= 790)**		**Mutation-positive cases (*n *= 37)**		**Mutation-positive cases vs. controls**		**Mutation-negative cases (*n *= 616)**		**Mutation-negative cases vs. controls**		**Mutation-positive vs. mutation-negative cases**	
	***n***	**(%)**	***n***	**(%)**	**OR**^a^	**95% CI**	***n***	**(%)**	**OR**^a^	**95% CI**	**OR**^a^	**95% CI**
**OC use**												
Never	319	(40.4)	14	(37.8)	1.00	---	224	(36.5)	1.00	---	1.00	---
Ever	470	(59.6)	23	(62.2)	1.72	0.66–4.44	390	(63.5)	1.15	0.87–1.52	1.53	0.61–3.84
**OC duration**												
Never	319	(40.4)	14	(37.8)	1.00	---	224	(36.5)	1.00	---	1.00	---
<5 years	232	(29.4)	8	(21.6)	1.08	0.35–3.38	194	(31.6)	1.27	0.94–1.73	1.01	0.33–3.06
5–10 years	162	(20.5)	8	(21.6)	1.75	0.53–5.77	123	(20.0)	0.95	0.67–1.35	1.79	0.59–5.43
>10 years	76	(9.6)	7	(8.9)	3.73	1.16–12.03	73	(11.9)	1.18	0.77–1.81	2.66	0.81–8.67
P_trend_						0.02				0.84		0.07
**Age at first use of OCs**												
Never	319	(40.4)	14	(37.8)	1.00	---	224	(36.6)	1.00	---	1.00	---
≤20 years	215	(27.2)	8	(21.6)	1.18	0.32–4.42	192	(31.4)	0.99	0.69–1.41	1.44	0.41–5.09
>20 years	255	(32.3)	15	(40.5)	1.81	0.71–4.63	196	(32.0)	1.20	0.89–1.60	1.56	0.61–3.96
**Age at last use of OCs**												
Never	319	(40.4)	14	(37.8)	1.00	---	224	(36.6)	1.00	---	1.00	---
≤30 years	273	(34.6)	10	(27.0)	1.18	0.36–3.90	226	(36.9)	1.10	0.80–1.52	1.39	0.45–4.25
>30 years	197	(25.0)	13	(35.1)	1.97	0.75–5.15	162	(26.5)	1.16	0.85–1.58	1.63	0.62–4.29
**Year of first OC use**												
Never	319	(40.4)	14	(37.8)	1.00	---	224	(36.6)	1.00	---	1.00	---
≤1975	389	(49.3)	19	(51.4)	1.72	0.67–4.43	292	(47.7)	1.15	0.87–1.52	1.54	0.61–3.89
>1975	81	(10.3)	4	(10.8)	1.51	0.28–8.12	96	(15.7)	0.98	0.62–1.55	1.58	0.35–7.13
**First use before first full-term pregnancy**												
No	233	(54.8)	11	(57.9)	1.00	---	169	(51.1)	1.00	---	1.00	---
Yes	192	(45.2)	8	(42.1)	0.66	0.15–2.93	162	(48.9)	1.12	0.76–1.66	0.78	0.18–3.31
**Recency of OC use (years since stopping)**												
Never	319	(42.3)	14	(37.8)	1.00	---	224	(39.4)	1.00	---	1.00	---
10+	374	(49.5)	17	(45.9)	1.70	0.65–4.43	278	(48.8)	1.15	0.86–1.52	1.52	0.59–3.92
<10	62	(8.2)	6	(16.2)	3.63	0.80–16.45	67	(11.8)	1.06	0.65–1.72	3.65	0.86–15.47
P_trend_						0.10				0.56		0.10
**OC duration and recency of use (years since stopping)**												
Never	319	(42.3)	14	(37.8)	1.00	---	224	(39.4)	1.00	---	1.00	---
<10, <10	31	(4.1)	2	(5.4)	1.43	0.19–10.77	32	(5.6)	0.84	0.44–1.58	3.04	0.46–20.06
<10, 10+	315	(41.7)	13	(35.1)	1.53	0.55–4.26	245	(43.1)	1.21	0.90–1.62	1.25	0.46–3.41
10+, <10	31	(4.1	4	(10.8)	6.49	1.32–31.89	35	(6.2)	1.32	0.73–2.38	3.53	0.73–17.09
10+, 10+	59	(7.8)	4	(10.8)	1.93	0.52–7.11	33	(5.8)	0.81	0.49–1.35	3.01	0.78–11.59
**Postmenopausal HRT use**												
Never	219	(50.3)	13	(59.1)	1.00	---	180	(61.0)	1.00	---	1.00	---
Ever	216	(49.7)	9	(40.9)	0.83	0.28–2.42	115	(39.0)	0.62	0.44–0.87	1.23	0.42–3.61
**Postmenopausal HRT duration**												
Never	219	(50.3)	13	(59.1)	1.00	---	180	(61.0)	1.00	---	1.00	---
<5 years	105	(24.1)	3	(13.6)	0.60	0.14–2.51	56	(19.0)	0.61	0.40–0.92	1.37	0.30–6.24
5+ years	111	(25.6)	6	(27.3)	1.07	0.31–3.70	59	(20.0)	0.64	0.42–0.96	1.17	0.35–3.91

^a^ORs adjusted for sampling fraction, age, race, menopausal status (where appropriate), first-degree family history of breast cancer, parity/age first full-term pregnancy, breast feeding (ever, never), smoking status (never, former, current), alcohol intake (ever, never), age at menarche (<11, 11–12, 13, 14+), and body mass index (<25, 25 to <30, 30+). CI, confidence interval; *ESR1*, estrogen receptor alpha; HRT, hormone replacement therapy; OC, oral contraceptive; OR, odds ratio.

Several ORs for OC use were elevated but were not statistically significant, probably due to the small number of mutation-positive tumors. The *P *values for trend among *ESR1 *A908G mutation-positive cases were 0.02 for duration of OC use (less than 5, 5 to 10, or more than 10 years) and 0.10 for recency of OC use (10 or more years or less than 10 years). In contrast to our findings in *ESR1 *mutation-positive cases, OC use, even long-term or recent use, was not associated with mutation-negative breast cancer. Among mutation-negative cases, the *P*_trend _values were 0.84 for duration of OC use (less than 5, 5 to 10, or more than 10 years) and 0.56 for years since stopping (10 or more years or less than 10 years). Other measures of OC use similarly showed no associations with *ESR1 *mutation-negative breast cancer.

Comparing mutation-positive to mutation-negative cases, OC use for more than 10 years and OC use within the past 10 years were both associated with *ESR1 *A908G mutation positivity, but these ORs were not statistically significant (OR = 2.66, 95% CI = 0.81 to 8.67 for use for more than 10 years; OR = 3.65, 95% CI = 0.86 to 15.47 for use within the past 10 years). ORs were suggestive of a positive trend, but the trend tests were not statistically significant.

Among postmenopausal women, there was no clear evidence for an association of HRT use with *ESR1 *A908G mutation-positive cancer (OR = 0.83, 95% CI = 0.28 to 2.42), but HRT use was inversely associated with mutation-negative breast cancer (OR = 0.62, 95% CI = 0.44 to 0.87 for ever users; OR = 0.64, 95% CI = 0.42 to 0.96 for duration of 5 or more years) (Table 2). We were unable to analyze HRT according to former and current use because the numbers in each case group were too sparse.

First-degree family history of breast cancer was associated with *ESR1 *mutation-positive breast cancer (OR = 2.54, 95% CI = 1.16 to 5.55) (Table 1). Further adjustment for OC use had little effect on this association (OR = 2.71, 95% CI = 1.16 to 6.33). These results suggest that OC use and family history of breast cancer may independently influence risk of the ESR1 mutation-positive subset of breast cancer.

## Discussion

Evidence is accumulating to suggest that breast cancer is a collection of biologically distinct disease subtypes characterized by unique gene expression profiles, molecular or protein markers, and that exhibit variable clinical behavior, prognosis, and response to therapies [9,10,18-21]. Similarly, data obtained from some epidemiologic studies of breast cancer suggest that tumor subsets classified according to certain somatic or protein expression changes may be associated with specific etiologic risk factors [17,22-26]. Consistent with this, our study has revealed that first-degree family history of breast cancer (or breast and/or ovarian cancer) may be a risk factor for breast tumors carrying the *ESR1 *A908G mutation. Recent (within 10 years) and long-term (more than 10 years) use of OCs may also be associated with mutation-positive breast cancer; however, due to small numbers, most of these results were not statistically significant. In contrast, mutation-negative breast cancer was not associated with OC use but instead was associated with several reproductive factors, including longer duration of ovarian function and younger age at menarche, which increase exposure to endogenous hormones. HRT use was inversely correlated with the mutation-negative breast cancer subtype, but due to the small number of postmenopausal cases carrying the mutation, an effect of HRT on *ESR1 *A908G mutation-positive breast cancer could not be adequately assessed.

Many hormonal or reproductive factors that increase exposure to estrogen and/or progesterone are risk factors for breast cancer [1], and the contribution of OC use to breast cancer development has been extensively researched. Recent and longer durations of OC use have shown the most consistently positive, though small, associations with breast cancer [27-35], observations which are compatible with our findings for the *ESR1 *A908G mutation-positive cases. The 1996 meta-analysis of the published data on breast cancer risk and hormonal contraceptives by the Collaborative Committee on Hormonal Factors in Breast Cancer, the most comprehensive assessment of the impact of OCs on breast cancer risk [30], found that current and recent users of combined OCs had a small increased risk of breast cancer which decreased with time such that no significant excess risk remained 10 years after last use. Breast tumors that developed among ever OC users were less likely to have distant metastases; however, other characteristics of the primary tumor were not specifically evaluated. Young age at first use of OCs and use before the first full-term pregnancy carry elevated risks for breast cancer in several studies [28,32,35]; however, we found only a small, non-significant association with later age at first use of OCs among the *ESR1 *A908G mutation-positive subgroup. Additionally, associations with OCs have been noted among some subsets of breast cancer cases, including younger women [27,28,35], those with a family history of breast cancer [36], germline mutations in *BRCA1 *or *BRCA2 *[37-39], or other genetic polymorphisms [40]. Case subgroups defined by certain tumor characteristics have been associated with OC use, including lobular or mixed lobular/ductal histology [41], and those expressing ER [26], HER2 [17], p53 [23], or cyclin D1 [24].

In our study, *ESR1 *A908G mutation-positive breast cancer was strongly associated with recent and long-term OC use even though there was only a small main effect of OC use in the CBCS [31]. This finding suggests that characterization of breast tumors according to *ESR1 *A908G mutation status may help to uncover a subgroup of women for whom OC use may be a stronger risk factor. Furthermore, the mutation-negative subgroup may be associated with certain risk factors that represent greater exposure to endogenous hormones. Li and colleagues [42] noted distinct effects of exogenous versus endogenous hormonal factors on tumor histology, with OC use being more strongly associated with the development of lobular and mixed lobular/ductal breast tumors whereas endogenous hormonal factors, including longer duration of ovarian function and higher BMI, were more strongly associated with risk of ductal breast cancer. Several other case-control studies have also found that use of combined HRT or OC was associated mainly with an increase in lobular and mixed lobular/ductal breast cancer [41,43,44]. The relationship between *ESR1 *A908G mutation-positive breast cancer and OC use is not surprising as the mutation-positive subgroup of tumors in the CBCS was more likely to have mixed lobular/ductal histology [14].

These results suggest the possibility that endogenous and exogenous hormones may be differentially associated with development of breast cancer characterized by the presence or absence of the *ESR1 *A908G mutation. The exact basis for this is unclear but could be related to qualitative and/or quantitative differences between contraceptive hormones and estradiol (and/or progesterone), the timing of exposure to OCs, or differences in the biological effects elicited by the mutant and wild-type receptors.

Exposure to OCs, particularly the estrogen component, increases proliferative activity in breast epithelium [45-50]. The *ESR1 *A908G mutant was reported to be hypersensitive to estrogen and was associated with cellular proliferation at sub-physiologic levels of estradiol *in vitro *compared with the wild-type receptor [11]. Additionally, the A908G mutation was originally detected in breast hyperplasias [11], which may be risk factors and, in some instances, early precursors of invasive breast cancer [51-53]. We have also detected this mutation in benign breast lesions that contain histologic features other than usual hyperplasia (K. Conway, unpublished data). Given the abnormal function described for this somatic *ESR1 *A908G variant and its presence in very early breast lesions, it is biologically plausible that exposure of breast tissue to exogenous hormones during the premenopausal years could be associated with the subgroup of breast tumors carrying this mutation. From a mechanistic standpoint, our results suggest that OCs could interact with the pre-existing *ESR1 *A908G mutant receptor in early pre-neoplastic breast lesions to stimulate epithelial proliferation, thus driving the accumulation of additional genetic errors leading to neoplasia. The possibility that such a common exposure as OC use might stimulate the outgrowth of cells carrying this somatic mutation makes the *ESR1 *A908G variant a potentially important candidate marker for studies of breast cancer etiology and progression.

Studies on combined estrogen and progestin HRT have generally found longer duration and recent use of HRT to be associated with increased risk of breast cancer [54]. HRT with estrogen alone or combined estrogen and progestin is associated with increased proliferation in normal breast tissue of postmenopausal women [55]. Postmenopausal HRT use was somewhat protective for the mutation-negative subgroup, similar to what was previously reported overall in the CBCS [56].

Compared with controls, breast cancer cases in the CBCS with *ESR1 *A908G mutation-positive tumors were more likely to have a first-degree family history of breast cancer whereas the mutation-negative cases were not; this finding was supported by case-case comparisons. This association is unlikely to be related to germline defects in *BRCA1 *or *BRCA2 *since 21 of the 37 *ESR1 *mutation-positive cases had previously been screened for mutations in these genes but none was found [57] (B Newman and M-C King, unpublished data). However, we cannot rule out the possibility that these cases may carry germline variations in other genes which could influence breast cancer susceptibility and which include variants within *ESR1 *that may be linked with mutation status. Several previous studies have evaluated the risk of breast cancer associated with OC use among women with a family history of breast cancer [33,40,58] or those carrying germline defects in *BRCA1 *or *BRCA2 *[37-39]. Grabrick and colleagues [36] suggested that OC use was more strongly associated with breast cancer among women with a family history, but at least two subsequent studies have not supported this finding [29,58]. However, in some studies, OC use has been found to increase breast cancer risk among women with known *BRCA1 *or *BRCA2 *germline variants [37-39].

The primary strengths of this study are the population-based case series comprised mainly of early stage tumors, the careful assessment of the *ESR1 *A908G mutation by means of a stringent screening algorithm, and the large sample size nearly half of which consisted of premenopausal women. Despite the large number of cases evaluated (*n *= 653), this study is limited by the small number of mutation-positive breast tumors identified. Therefore, our results should be interpreted with caution. However, the significant findings on OC use and *ESR1 *A908G mutation-positive breast cancer warrant further study in larger data sets.

## Conclusion

Characterization of breast tumors for the *ESR1 *A908G point mutation, shown by Fuqua and colleagues [9] to be hypersensitive to estrogen, may reveal important etiologic clues. *ESR1 *A908G mutation-positive breast cancer was significantly associated with longer duration and recent use of OCs and with a first-degree family history of breast cancer, suggesting that OCs may interact with the *ESR1 *A908G mutant receptor in the development of some breast tumors. Some reproductive factors linked to greater exposure to endogenous hormones, including younger age at menarche and longer duration of ovarian function, were associated with the mutation-negative subgroup, suggesting that endogenous hormonal factors may be more important for mutation-negative cancer. Additional studies will be required to confirm these findings.

## Abbreviations

AFFTP = age at first full-term pregnancy; BMI = body mass index; CBCS = Carolina Breast Cancer Study; CI = confidence interval; ER = estrogen receptor; *ESR1 *= estrogen receptor alpha; FFPE = formalin-fixed paraffin-embedded; HRT = hormone replacement therapy; OC = oral contraceptive; OR = odds ratio.

## Competing interests

The authors declare that they have no competing interests.

## Authors' contributions

EP and DT conducted the laboratory analyses. C-KT conducted the statistical analyses. KC, RCM, BN, and PM participated in the interpretation of results and writing of the manuscript. SNE conducted the laboratory analyses and participated in the interpretation of results and writing of the manuscript. All authors read and approved the final manuscript.
